# *Primulinasilaniae* sp. nov. (Gesneriaceae) from the limestone area of Guizhou Province, China

**DOI:** 10.3897/phytokeys.185.72099

**Published:** 2021-11-26

**Authors:** Jin-Quan Zhang, Hong Huang, Mei-Jun Li, Mei Huang, Quan-Yuan Li, Yu-Lu Zhou, Yi Chen, Fang Wen, Xin-Xiang Bai

**Affiliations:** 1 Forestry College, Guizhou University, CN-5500252 Guiyang, China Guizhou University Guiyang China; 2 Guangxi Key Laboratory of Plant Conservation and Restoration Ecology in Karst Terrain, Guangxi Institute of Botany, Guangxi Zhuang Autonomous Region and Chinese Academy of Sciences, CN-541006 Guilin, China Guangxi Institute of Botany, Guangxi Zhuang Autonomous Region and Chinese Academy of Sciences Guilin China; 3 Gesneriad Committee of China Wild Plant Conservation Association, National Gesneriaceae Germplasm Resources Bank of GXIB, Gesneriad Conservation Center of China (GCCC), CN-541006 Guilin, Guangxi, China National Gesneriaceae Germplasm Resources Bank of GXIB, Gesneriad Conservation Center of China Guilin China

**Keywords:** flora of Guizhou, karst, new taxon, taxonomy, subtribe, Didymocarpinae

## Abstract

*Primulinasilaniae* X.X.Bai & F.Wen, a new species of *Primulina* Hance (Gesneriaceae) from the limestone area of Wangmo County, Guizhou Province, is described and illustrated. The new species is similar to *P.spiradiclioides* Z.B.Xin & F.Wen, but can be easily distinguished from the latter by a combination of characteristics, especially in the lateral veins of its leaf and floral shape and tube. At present, three populations in one locality of this new taxon were found, totaling about 600 mature individuals. According to the IUCN Red List Categories and Criteria (Version 3.1), the species is provisionally assessed as Vulnerable [VU D1].

## Introduction

*Primulina* Hance (Gesneriaceae, Didymocarpoideae, Trib. Didymocarpoideae, subtrib. Didymocarpinae) was a monotypic genus until 2011 (Wang et al. 2011; Weber et al. 2011). It was first described and published in 1883 by Henry Fletcher Hance, a British botanist and the Vice Consul for Foreign Affairs of Huangpu Port in Guangdong (Hance 1883). The type species of this genus is *P.tabacum* Hance, which once was considered a rare cave plant in limestone montane areas and collected in Lianzhou City, Guangdong Province, China (Wang et al. 2013). After 2011, plant taxonomists revised the classification system of *Chirita* and its related genera based on molecular and morphological evidence. Now, as a result of this reorganization and newly described species, the newly defined *Primulina* has become the most diverse genus of Gesneriaceae in China (Li and Wang 2007; Möller et al. 2009, 2011; Wang et al. 2011; Weber et al. 2011). As of October 2021, there were 219 species (excluding infraspecific taxa) (GRC 2021), and most of the species in this genus were discovered from Guangxi, China (Wei 2018).

Although *Primulina* has only been found in southern and southwestern China and northern Vietnam, mainly in limestone areas (Möller et al. 2016), there is still great potential to find undescribed species diversity (Möller 2019). As the main locality of differentiation and diversity of Gesneriaceae in China, many new taxa have been discovered or reported in Guizhou (Xu et al. 2017). From 2019 to October 2021, 21 new species (including infraspecific taxa) of the genus *Primulina* have been described (Wen et al. 2021), including two species from Guizhou Province *P.serrulata* R.B.Zhang & F.Wen from Rongjiang County (Hong et al. 2019) and *P.flexusa* F.Wen, Tao Peng & B.Pan (Peng et al. 2020) from Duyun City (Wen et al. 2021).

In July 2020, when we investigated plants in Wangmo County, we found a plant on moist, shady limestone cliffs that appeared to represent an undescribed species of *Primulina* based on its old fruits and leaves. After regular observation, we photographed the flowers and collected the specimens in November 2020. Comparing these specimens and living plant materials with the type specimens and protologues of all 219 species of *Primulina*, we found that these specimens and plants neither fit the existing protologues nor conformed to the type specimens of these species. Nevertheless, the shape and texture of leaves are most similar to *P.spiradiclioides* Z.B.Xin & F.Wen (Xin et al. 2020); it can be distinguished by a combination of several morphological characters of the leaf margin, lateral veins, leaf indumentum, and floral features. Thus, we confirmed that it represented a new species of *Primulina* and describe and illustrate it here.

## Materials and methods

The plant material for description was collected in the field at its type locality in 2020. Morphological observations and dissections of plant material of this new species were made under a stereoscopic microscope (Olympus SZ61, Tokyo, Japan) and measured and described using the terminology used by Wang et al. (1998). Electronic specimens stored in herbaria in China, Vietnam, the United States, and the United Kingdom (e. g. E, GH, HN, IBK, K, IBSC, HITBC, KUN, MO, PE, PH, US, and VNMN) were examined.

## Taxonomic treatment

### Primulina silaniae

Taxon classificationPlantaeLamialesGesneriaceae

X.X.Bai & F.Wen sp. nov.

3CE6A311-D765-58F0-8E48-E3565C0CE967

urn:lsid:ipni.org:names:77233712-1

Figures 1 , 2

#### Diagnosis.

The new species is similar to *Primulinaspiradiclioides*, but can be easily distinguished from the latter by leaf margin entire with occasionally a few long hairs, glabrous on adaxial and abaxial surfaces (*vs.* margin serrate and not villous, lamina adaxial and abaxial surfaces densely whitish villous), no lateral veins on adaxial and abaxial surfaces (*vs.* inconspicuous on adaxial surfaces, distinctly raised on abaxial surface), pedicels 8–31 mm long (*vs.* ca. 5 mm), calyx inside glabrous (*vs.* sparsely pubescent), bluish-purple corolla lobes, with two short brownish-yellow stripes in the white throat (*vs.* corolla mouth white, forming a conspicuous pentagon), corolla tube slightly curved (*vs.* straight), ovary ca. 6mm long (*vs.* 2–2.5 mm), style 19–22 mm long (*vs.* ca. 2.5 mm), capsule, 8–9 mm long (*vs.* 10–15 mm).

**Figure 1.**
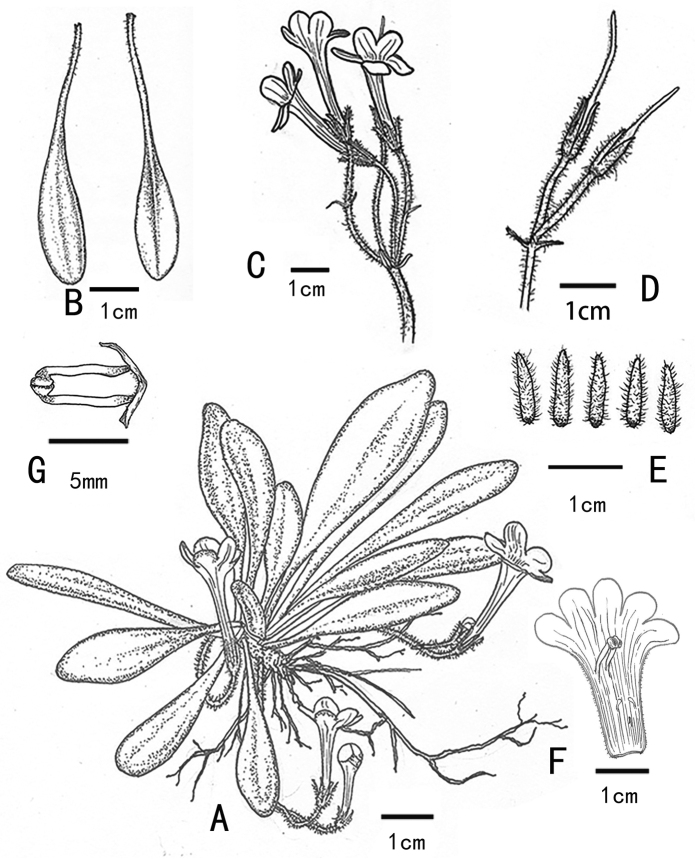
*Primulinasilaniae* X.X.Bai & F.Wen, sp. nov. **A** habit **B** adaxial (left) and abaxial (right) surfaces of leaves **C** flowering cyme **D** young capsules **E** dissected calyx lobes **F** half opened corolla cover, showing stamens **G** stamens with cohering anthers. Drawn by Yi Chen from the holotype.

#### Type.

CHINA, Guizhou Province: Wangmo County, Sanglang Town, 25°13'N, 106°26'E, altitude ca. 564 m, November 28, 2020, *Xin-Xiang Bai* et al., *BXX20201128-01* (Holotype: GZAC!; Isotype: GZAC!; IBK!)

**Figure 2.**
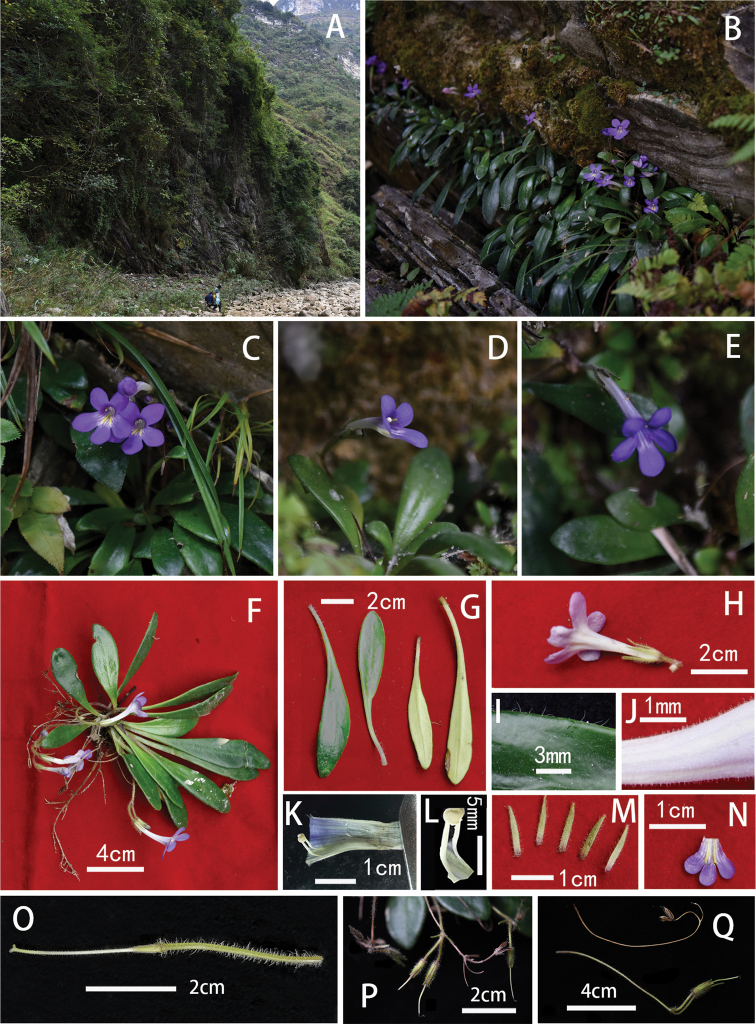
*Primulinasilaniae* X.X.Bai & F.Wen, sp. nov. **A, B** habitat **C** front view of flower **D, E** oblique side view of flowers **F** plant **G** adaxial (left two) and abaxial (right two) surfaces of leaves **H** corolla **I** partial, enlarged leaf **J** partial, enlarged corolla tube **K** stamen **L** coherent anthers **M** dissected calyx lobes **N** lower distal part of corolla **O** pistil, pedicel and calyx with sepals removed **P, Q** dehisced and immature fruits. Photographs by Xin-Xiang Bai.

#### Description.

Herb perennial, lithophytic. Rhizomatous stem cylindrical, 0.9–1.2 cm long, ca. 0.5 cm in diameter. Leaves basal, fleshy and brittle, 8–18, crowded at the apex of stem; petiole occasionally villous, slightly concave on adaxial surface, 1–4 × 0.2–0.4 cm. Leaf blade oblong or oblanceolate, adaxially dark green, abaxially light green, 4–7 × 0.8–1.0 cm, glabrous on adaxial and abaxial surfaces, margin entire, occasionally a few long hairs, base tapering to petiole, apex acuminate to obtuse; midrib inconspicuous on adaxial surface, conspicuous on abaxial surface, no lateral veins. Cymes axillary, 1–4, 1–4–flowered; peduncle 3.2–12.5 cm long, ca. 1 mm in diameter, densely white pubescent; bracts 2, opposite, oblong, ca. 2.5 × 1 mm, adaxially pubescent, margin entire, apex acute; bracteoles ca. 2 × 0.5 mm, same color and indumentum as bracts; pedicels 8–31 mm long, ca. 0.8 mm in diameter, densely white pubescent; calyx 5-lobed, free from base, lobes equal, pale green, lanceolate to narrowly linear, ca. 9 × 1.5 mm, outside densely white pubescent, inside glabrous, margin entire, apex acute. Corolla infundibuliform, bluish-purple lobed, 3.3–3.6 cm long, two short brownish-yellow stripes with pubescent in white throat of the corolla; tube white, slender tubular, slightly curved, 2.8–3.1 cm long, base ca. 0.2 cm in diameter, mouth ca. 0.4 cm in diameter, tube outside sparsely pubescent in upper parts, lower parts and inside glabrous; limb distinctly 2-lipped, adaxial lip 2-lobed, lobes rounded, margin entire, ca. 6 × 5 mm; abaxial lip 3-lobed, lobes oblong, ca. 9 × 6 mm. Stamens 2, adnate ca. 16 mm above the base of the corolla tube; filaments ca. 6 mm long, terete; anthers coherent by entire adaxial surfaces, elliptic, ca. 2 × 1.5 mm, pale yellow, glabrous; staminodes 3, white, lateral ones ca. 2.1 mm long, adnate ca. 8 mm above the base of the corolla tube, terete, the middle one ca. 1.2 mm long, adnate to ca. 8 mm above the base of the corolla tube. Disk annular, yellow-green, margin entire, ca. 1 mm high. Pistils, ca. 2.6 cm long; style white, linear, 19–22 mm long, densely glandular-pubescent; ovary yellowish-green, ca. 6 mm long, glandular-pubescent. Stigma apex slightly 2-lobed; ca. 2 mm long, ca. 1 mm wide, Capsule narrowly cylindrical, longitudinal dehiscence, 8–9 mm long, ca. 3.5 mm in diameter; calyx and style persistent, white pubescent outside.

#### Etymology.

The epithet ‘silaniae’ is coined to honour Prof. Si-Lan Dai, the famous horticulturist at the Beijing Forestry University. She is also the former supervisor of one of the authors, Prof. Xin-Xiang Bai. Meanwhile, one of the given names of Prof. Si-Lan Dai, namely ‘Lan’, shares the same pronunciation in Chinese with the color blue. Thus, the scientific name also hints at the bluish to purplish-blue corolla of this new taxon.

#### Vernacular name.

Sī Lán Bào Chūn Jù Tái (Chinese pronunciation); 思兰报春苣苔 (Chinese name).

#### Phenology.

Flowering from November to February of the following year, fruiting from March to May.

#### Distribution and habitat.

The species has only been found in Wangmo County, the type locality. It grows on moist, shady limestone cliffs at altitudes of 550 to 570 meters.

#### Provisional IUCN conservation assessment.

At present, *Primulinasilaniae* is only found in the type locality. There are three populations with ca. 600 matures individuals, all of which grow on moist and shady limestone cliffs. One of the populations has a small number of mature individuals and is located by the roadside, easily disturbed by human activities. It is therefore assessed as Vulnerable [VU D1] according to the IUCN Red List Categories and Criteria (Version 3.1) (IUCN 2012, 2019).

#### Additional specimens examined.

Paratypes. CHINA Guizhou Province: Wangmo County, Sanglang Town, 25°13'N, 106°26'E, 416 m, a.s.l., 22 December 2020, *Xin-Xiang Bai et al*., *BXX20201122-01* (GZAC!); The same locality, 11 April 2021, *Xin-Xiang Bai et al.*, *BXX20210411-01* (GZAC!); The same locality, 1 November 2020, *Xin-Xiang Bai et al., BXX20201101-01* (GZAC!).

#### Notes.

The leaf of *Primulinasilaniae* is fleshy and brittle, glabrous on both sides, with an entire margint that occasionally has a few long hairs, base tapering to petiole, apex acuminate to obtuse; midrib inconspicuous on adaxial surface, conspicuous on abaxial surface, no lateral veins. These characteristics differ from those of other *Primulina* species and can be clearly distinguished from *Primulinaspiradiclioides* in morphological characteristics (Table 1).

**Table 1.** Detailed comparisons of *Primulinasilaniae* and *P.spiradiclioides*.

**Characters \ species**	***P.silaniae***	***P.spiradiclioides***
Leaf-blades	margin entire, occasionally a few long hairs	margin serrate, densely whitish villous
Lateral veins	inconspicuous on adaxial and abaxial surfaces	inconspicuous on adaxial surfaces, distinctly raised on abaxial surface
Leaf indumentum	glabrous on both sides	adaxial and abaxial surfaces densely whitish villous
Pedicels length	8–31 mm	ca. 5 mm
Calyx indumentum	inside glabrous	inside sparsely pubescent
Corolla	bluish-purple lobes, two short brownish-yellow stripes in white throat of corolla	corolla mouth white, forming a conspicuous pentagon
Corolla tube	slightly curved	straight
Ovary length	ca. 6 mm	2–2.5 mm
Style length	19–22 mm	ca. 2.5 mm
Capsule length	8–9 mm	10–15 mm
Stamen insertion	adnate ca. 16 mm above corolla tube base	adnate ca. 1.7 mm above corolla tube base
Calyx indumentum	inside glabrous	inside sparsely pubescent

## Supplementary Material

XML Treatment for Primulina silaniae
